# An optimized FusX assembly-based technique to introduce mitochondrial TC-to-TT variations in human cell lines

**DOI:** 10.1016/j.xpro.2022.101288

**Published:** 2022-04-14

**Authors:** Bibekananda Kar, Ankit Sabharwal, Santiago Restrepo-Castillo, Brandon W. Simone, Karl J. Clark, Stephen C. Ekker

**Affiliations:** 1 Department of Biochemistry and Molecular Biology, Mayo Clinic, Rochester, MN, USA; 2 Mayo Clinic Graduate School of Biomedical Sciences, Virology and Gene Therapy Track, Mayo Clinic, Rochester, MN, USA

**Keywords:** Cell Biology, Genetics, Sequencing, Molecular Biology, CRISPR

## Abstract

The FusX TALE Based Editor (FusXTBE) is a programmable base editing platform that can introduce specific TC-to-TT variations in the mitochondrial DNA (mtDNA). Here, we provide a protocol describing the synthesis and testing of the FusXTBE plasmids in cultured human cell lines. This tool is designed to be easily modified to work in diverse applications where editing of mitochondrial DNA is desired.

For complete details on the use and execution of this protocol, please refer to Sabharwal et al. (2021) and Ma et al. (2016).

## Highlights

• Optimized protocol to introduce specific TC-to-TT edits in the mtDNA
• Generation of FusXTBE arms and validation of TC-to-TT editing
• TALE Writer program can be used to map potential mtDNA base editing sites
• FusXTBE arms design can be automated with TALE Writer

## Graphical abstract

**Figure:**
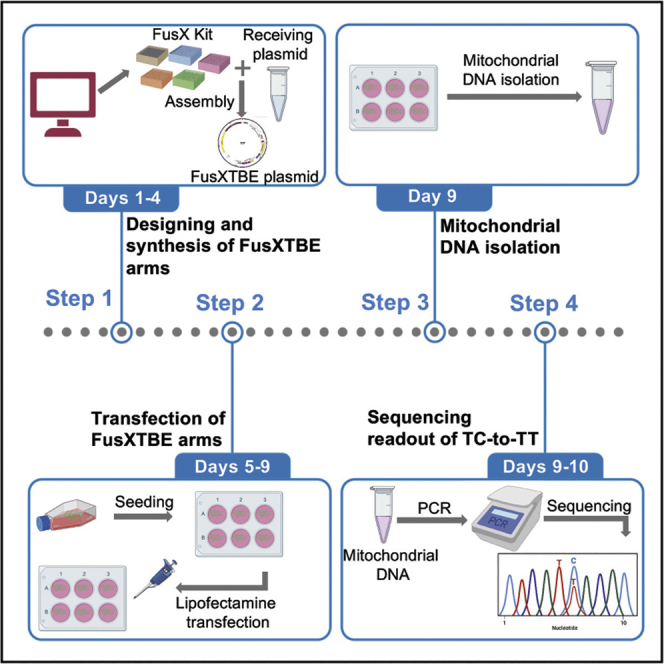

## Teaser

The FusX TALE Based Editor (FusXTBE) is a programmable base editing platform that can introduce specific TC-to-TT variations in the mitochondrial DNA (mtDNA). Here, we provide a protocol describing how to synthesize the FusXTBE plasmids. We detail steps to test the FusXTBE editing efficiencies and to validate TC-to-TT editing. The protocol has been tested using immortalized cell lines (e.g., HEK293T and HT1080O and in zebrafish; however, the tool can be modified to work in diverse applications for editing mtDNA.

## Before you begin

**Figure 1:**
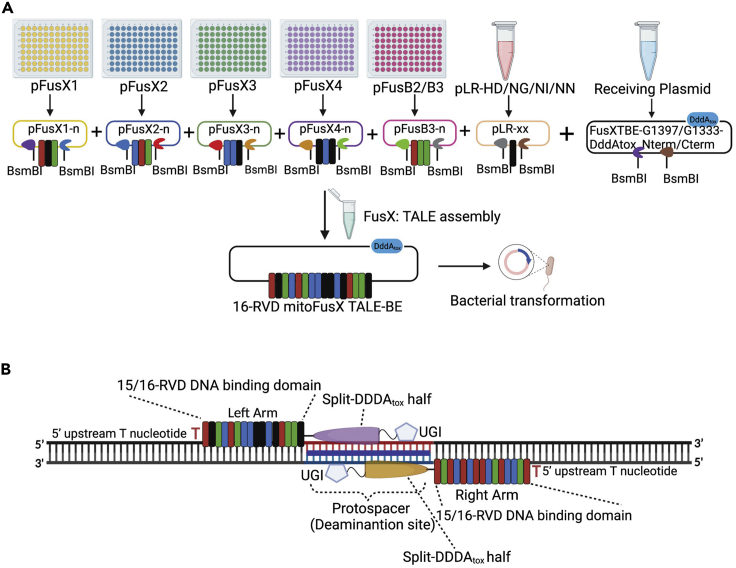
Schematic diagram of FusXTBE pairs used in this protocol and their assembly (A) Golden Gate cloning of FusX library enables the rapid synthesis of FusXTBE clones. (B) Each FusXTBE arm consists of a DNA binding domain with either 15 or 16-RVDs targeting 15 or 16-nucleotides DNA binding sequence, which is preceded by a 5′ T nucleotide. Attached to this module is a split half of the DddA_tox_ protein and an UGI molecule. The 14–18 bp protospacer region in between two arms are amenable for deaminase activity. Both the figures were created by using Biorender.com.

Transcription activator-like effectors (TALEs) are modular DNA-binding proteins that can be attached to various effector domains to manipulate the DNA (Bogdanove and Voytas, 2011). We use the FusX assembly system for the rapid and accurate assembly of TALEs to target both nuclear and mitochondrial DNA (Ma et al., 2016; Campbell et al., 2018, Sabharwal et al., 2021). In this protocol, we describe the synthesis of FusX-compatible programmable mtDNA base editors (FusXTBE) (Figure 1A). The mitochondrially directed FusXTBE system employs two arms of TALEs to introduce the base edit (TC-to-TT) in the protospacer sequence (space between two FusXTBE arms) (Figure 1B) (Sabharwal et al., 2021). Each arm in the FusXTBE architecture consists of a TALE domain comprising of an N-terminus, a DNA binding domain, and a C-terminus, fused to a cytidine deaminase DddA_tox_. DddA_tox_ is a toxin from *Burkholderia cenocepacia* and shows dsDNA cytidine deaminase activity (Mok et al., 2020).

The DddA_tox_ molecule is reported to be toxic when expressed as a single molecule, therefore an obligate two-component version was designed to facilitate base editing. This molecule has been split into two halves at either the G1397 or G1333 amino acid position. Targeted deamination occurs only when the two components form a functional complex, implemented when both halves are expressed as fusions to individual TALE arms (Mok et al., 2020). Each protospacer can be tested by four different combinations of FusXTBE arms (Table 1). We recommend trying both the orientations of any one of the split variants first before trying the other one.

**Table 1:** Orientations of FusXTBE arms for the TC-to-TT editing

Orientation	Left arm binding FusXTBE	Right arm binding FusXTBE
1	FusXTBE-Left arm-G1397-DddA_tox__Nterm	FusXTBE-Right arm-G1397-DddA_tox__Cterm
2	FusXTBE-Left arm-G1397-DddA_tox__Cterm	FusXTBE-Right arm-G1397-DddA_tox__Nterm
3	FusXTBE-Left arm-G1333-DddA_tox__Nterm	FusXTBE-Right arm-G1333-DddA_tox__Cterm
4	FusXTBE-Left arm-G1333-DddA_tox__Cterm	FusXTBE-Right arm- G1333-DddA_tox__Nterm

This technology has the potential to correct mitochondrial pathogenic point mutations, generate mitochondrial disease models, prematurely terminate protein translation, study mitochondrial biology, among other applications. This protocol explains how to introduce TC-to-TT mutations in mtDNA with a focus on immortalized cells. This approach can be readily used in different cell systems and *in vivo* models.

### Key considerations

The FusXTBE system has a strong sequence preference for the presence of T at the 5′ end of the target C (5′-TC). Moreover, both cytosines in 5′-TCC motifs are amenable to editing. In the target strand, base editing at the “5′-TC” motif results in “5′-TT”, consequently changing the corresponding “5′-GA” motif to a “5′-AA” motif in the opposite strand. The FusXTBE arms can bind either 15 or 16 bp on mtDNA and their binding regions must have an upstream 5′-T nucleotide for optimal binding (Figure 1B). The length of the protospacer should be in between 14-18 bp for optimal editing activity. Editing can happen at either strand of the protospacer region. A uracil DNA glycosylase inhibitor (UGI) domain was fused to the C-terminus of the DddA_tox_ halves for optimal editing outcomes. Before starting FusXTBE synthesis, we recommend amplifying the region around the protospacer and sequencing the PCR product to ensure that the target cells have matching binding sequences without any nucleotide polymorphisms. Even single nucleotide polymorphisms (SNPs) will greatly reduce or eliminate FusXTBE activity.

### FusX kit and receiving plasmids

The FusX assembly kit is available for ordering through the Ekker Lab Addgene inventory (Kit no. 1000000063) (https://www.addgene.org/kits/ekker-fusx/). The kit is shipped as four 96-well plates of bacterial glycerol stocks. It comprises of 336 glycerol stocks named as pFusX1 1–64, pFusX2 1–64, pFusX3 1–64, pFusX4 1–64, pFusB2 1–16, and pFusB3 1–64. You will also require four different 0.5-mer pLR plasmids. These can be ordered from the Voytas Lab Addgene inventory (https://www.addgene.org/Daniel_Voytas/). The four different pLR plasmids are: pLR-HD (https://www.addgene.org/30984/), pLR-NG (https://www.addgene.org/30995/), pLR-NI (https://www.addgene.org/31006/), and pLR-NN (https://www.addgene.org/31017/). Upon arrival of the kit components, the glycerol stocks should be streaked on spectinomycin-containing LB agar plates. Subsequently, grow each culture in spectinomycin-containing LB liquid media to isolate the individual plasmids and make glycerol stocks for future use. The maps of all plasmids are available on Addgene. Optionally, each plasmid can be sequence-verified using the universal M13F primer for Sanger sequencing. In this protocol, the following receiving plasmids are used: FusXTBE-G1397-DddA_tox__Nterm, FusXTBE-G1397-DddA_tox__Cterm, FusXTBE-G1333-DddA_tox__Nterm, FusXTBE-G1333-DddA_tox__Cterm. These receiving plasmids are available upon request and are currently in submission to Addgene. The plasmid maps of these receiving plasmid are provided as Supplemental material (Data S1, receiving plasmid maps, related to step 5). To improve FusXTBE assembly efficiency, it is recommended to use fresh aliquots of miniprepped receiving plasmids.

## Key resources table

**Table:** 

REAGENT or RESOURCE	SOURCE	IDENTIFIER
**Bacterial and viral strains**		

DH5α Competent Cells	Prepared in the lab	N/A

**Experimental models: Cell lines**		

HEK293T	ATCC	ACS-4500
HT1080	ATCC	CCL-121

**Chemicals, peptides, and recombinant proteins**		

Agarose	VWR Life Science	Cat#97062-250
Ethanol	Sigma-Aldrich	Cat#459844-500ML
*N,N*-Dimethylformamide	Sigma-Aldrich	Cat#227056-100ML
DMEM	Gibco	Cat#11965092
MEM	Corning	Cat#10-010-CM
FBS	Gibco	Cat#26140-079
Penicillin-Streptomycin	Gibco	Cat#15070-063
DPBS	Gibco	Cat#14190-144
0.25% Trypsin-EDTA	Gibco	Cat#25200–072
Lipofectamine 3000	Thermo Fisher Scientific	Cat#L3000001
Opti-MEM	Gibco	Cat#31985062
T4 DNA Ligase	New England BioLabs	Cat#M0202S
BsmBI-v2	New England BioLabs	Cat#R0739S
Esp3I	Thermo Fisher Scientific	Cat#ER0451
SphI	New England BioLabs	Cat#R3182S
XbaI	New England BioLabs	Cat#R0145S
ATP	Thermo Fisher Scientific	Cat#R0441
T4 DNA Ligase Buffer	New England BioLabs	Cat#B0202S
NEBuffer r3.1	New England BioLabs	Cat#B6003S
rCutSmart Buffer	New England BioLabs	Cat#B6004S
Gel Loading Dye, Purple	New England BioLabs	Cat#B7024S
HyperLadder 1kb	Meridian Bioscience	Cat#BIO-33053
Kanamycin sulfate	Sigma-Aldrich	Cat#K4000
Spectinomycin dihydrochloride pentahydrate	Sigma-Aldrich	Cat#S4014
LB broth	Fisher Scientific	Cat#BP1426-500
Agar	BD Biosciences	Cat#VWR 90000-762
Plasmid-Safe ATP-Dependent DNase	Lucigen	Cat#E3101K
MyTaq™ Red DNA Polymerase	Meridian Bioscience	Cat# BIO-21108
Q5 High-Fidelity DNA Polymerases	New England BioLabs	Cat#M0492S

**Critical commercial assays**		

QIAprep Spin Miniprep Kit	QIAGEN	Cat#27106
QIAquick Gel Extraction Kit	QIAGEN	Cat#28706
QIAquick PCR Purification Kit	QIAGEN	Cat#28106
DNeasy Blood & Tissue Kit	QIAGEN	Cat#69506

**Oligonucleotides**		

Forward primer for colony PCR and sanger sequencing (TAL-F1): TTGGCGTCGGCAAACAGTGG	Ma et al., 2016	N/A
Reverse primer for colony PCR and sanger sequencing (TAL-R1): GGCGACGAGGTGGTCGTTGG	Ma et al., 2016	N/A
Forward primer for MT-ND4 locus amplification and sanger sequencing (ND4-F1): GCCATTCTCATCCAAACC	Sabharwal et al., 2021	N/A
Reverse primer for MT-ND4 locus amplification and sanger sequencing (ND4-R1): GGTTGAGGGATAGGAGGAG	Sabharwal et al., 2021	N/A

**Recombinant DNA**		

FusX assembly kit	Ma et al., 2016	Addgene kit Cat#1000000063
pLR-HD	Cermak et al., 2011	Addgene plasmid Cat#30984
pLR-NG	Cermak et al., 2011	Addgene plasmid Cat#30995
pLR-NI	Cermak et al., 2011	Addgene plasmid Cat#31006
pLR-NN	Cermak et al., 2011	Addgene plasmid Cat#31017

**Software and algorithms**		

SnapGene	SnapGene	https://www.snapgene.com/
GraphPad Prism 9	GraphPad Software	https://www.graphpad.com/scientific-software/prism/
TALE Writer	Ekker Lab	https://colab.research.google.com/github/srcastillo/TALE-Writer/blob/main/TALE_Writer_Colab.ipynb
EditR	Moriarity Lab	http://baseeditr.com/

**Other**		

Membrane Filter, 0.22 μm pore size	Millex	Cat#SLGVM33RS
Plastic syringes	Fisher Scientific	Cat#22-652-090
0.2 μm PVDF syringe filter	MilliporeSigma	Cat#SLGV033RS
4.5 mm plating Beads	Zymo Research	Cat#S1001
Hemacytometer	Fisher Scientific	Cat#0267110
Nuclease-Free Water	Thermo Fisher Scientific	Cat#AM9937
150 × 15 mm petri dishes	Corning	Cat#351029
50 mL falcon tubes	Corning	Cat#430290
15 mL falcon tubes	Corning	Cat#352097
1.7 mL microcentrifuge tubes	GeneMate	Cat#VWR 490004-436
0.2 mL PCR tubes	GeneMate	Cat#VWR 490003-706
NanoDrop spectrophotometer	Thermo Fisher Scientific	Cat#ND1000
Microscope	Olympus	Cat#CKX41
Receiving plasmid maps	Mendeley Data: https://doi.org/10.17632/pw24c8ndk3.2	N/A
FusXTBE Builder Template	Mendeley Data: https://doi.org/10.17632/pw24c8ndk3.2	N/A

## Materials and equipment

### Prepare medium and stock solution

#### Preparation of LB liquid media

Add 25 g of commercial LB broth powder to 950 mL of water, dissolve it, bring the volume to 1 L and autoclave at 121°C for 20 min in a liquid cycle. Make aliquots of LB liquid media and store at 4°C for up to 6 months. Bring the aliquots to room temperature (∼25°C) and add the antibiotics before setting up the primary culture.

***Note:*** For this study, the final concentration of kanamycin and spectinomycin is 50 μg/mL.

#### Preparation of LB agar plates

To prepare LB agar plates, add 15 g of agar powder to 1 L of LB liquid media and autoclave at 121°C for 20 min in a liquid cycle. Allow the media to reach ∼50°C–55°C temperature and then add antibiotics, mix well, and pour approximately 25 mL into individual petri dishes. Allow the media to solidify at room temperature (∼25°C). LB agar plates can be stored at 4°C for up to 60 days.

#### Preparation of 0.3 M IPTG

Dissolve 714.93 mg of IPTG in 8 mL of Nanopure water and bring the volume up to 10 mL with Nanopure water. Filter sterilize using a 0.22 μm filter. Prepare aliquots in 1.5 mL microcentrifuge tubes and store at −20°C until future use.

#### Preparation of 20 mg/mL X-Gal

Dissolve 200 mg of X-Gal up to 8 mL of *N,N*-Dimethylformamide and bring the volume up to 10 mL with *N,N*-Dimethylformamide. Prepare aliquots in amber glass/polypropylene vials and store at −20°C until future use.

**CRITICAL:** Kanamycin, spectinomycin, IPTG and X-Gal are harmful if inhaled/ingested and are potential skin irritant too. Wear gloves while handling them.

## Step-by-step method details

### Designing and synthesis of FusXTBE arms

**Timing: ∼4 Days**

To edit a target cytosine residue, we employ dual chain FusXTBE arms (left and right arms binding to the forward and reverse strands, respectively). In this protocol, we describe how to design the FusXTBE arms, which bind target DNA sequences flanking the protospacer region. Once the FusXTBE arms are designed, we will describe the process of synthesizing them. As an example, to demonstrate FusXTBE-mediated base editing in human cells, we introduced a point mutation by changing a TGA codon to a TAA codon in the *MT-ND4* gene which encodes for NADH subunit 4. This protein is part of the mitochondrial enzyme complex I (Mimaki et al., 2012). The protospacer length, target cytosine position, and split orientation are all determinants of the base editing efficiency of split-DddA_tox_. The human mitochondrial sequence information can be retrieved from the “MITOMAP” server (https://www.mitomap.org/MITOMAP).

1. Determining the target sequence.

The first step is to identify the target “cytosine” residue on the mitochondrial genome that we want to edit to “thymine”. Some of the utilities of base editing can be to introduce a premature termination codon (PTC) in an ORF, revert a pathogenic T-to-C mutation, generate *in vitro/in vivo* disease model, and generate mutant proteins. Therefore, we need to choose the target “cytosine” nucleotide accordingly.

**Figure 2:**
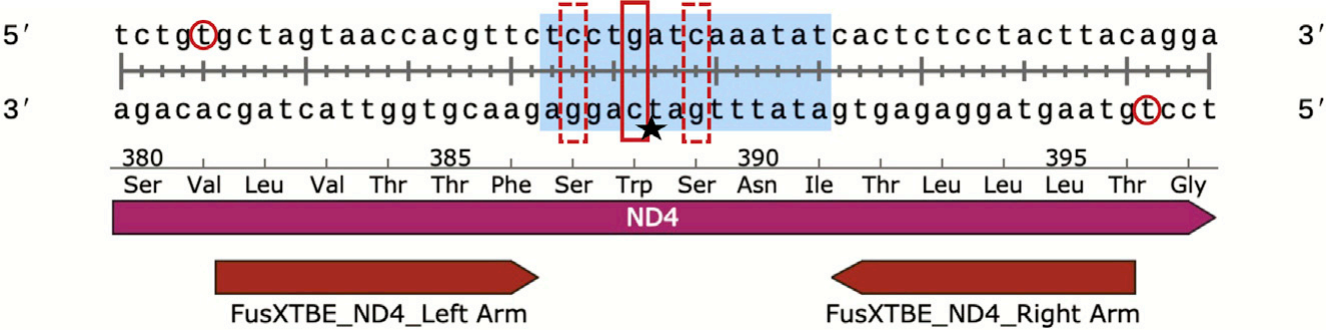
Schematic presentation of the design of the FusXTBE arms to target *MT-ND4* locus Within the protospacer (blue highlighted), the target cytosine nucleotide is highlighted within the solid square. The dotted squares represent the cytosine nucleotides which are amenable for base editing but not desired. Star mark indicates the presence of 5′ T nucleotide before target cytosine. Two FusXTBE arms are represented by the arrows design and the circles represent the 5′ T nucleotide upstream of the left and right binding regions.

Example: Recently, we targeted the human *MT-ND4* gene to introduce a premature stop codon (Sabharwal et al., 2021). The position of the target cytosine on human mtDNA was m.11922 and was present in the antisense strand, preceded by a thymine nucleotide. C-to-T transition at m.11922 position results in G-to-A on the forward strand, thereby introducing a stop codon [TGA (W)>TAA (stop)] in the ORF of *MT-ND4*. We selected the 14 bp (TCCTGATCAAATAT), spanning from m.11918 to m.11931 as the “target protospacer” (Figure 2). Within the protospacer, two other cytosine residues at m.11919 and m.11925 were amenable to C-to-T editing (undesired in the context of our experiment).

**Figure 3:**
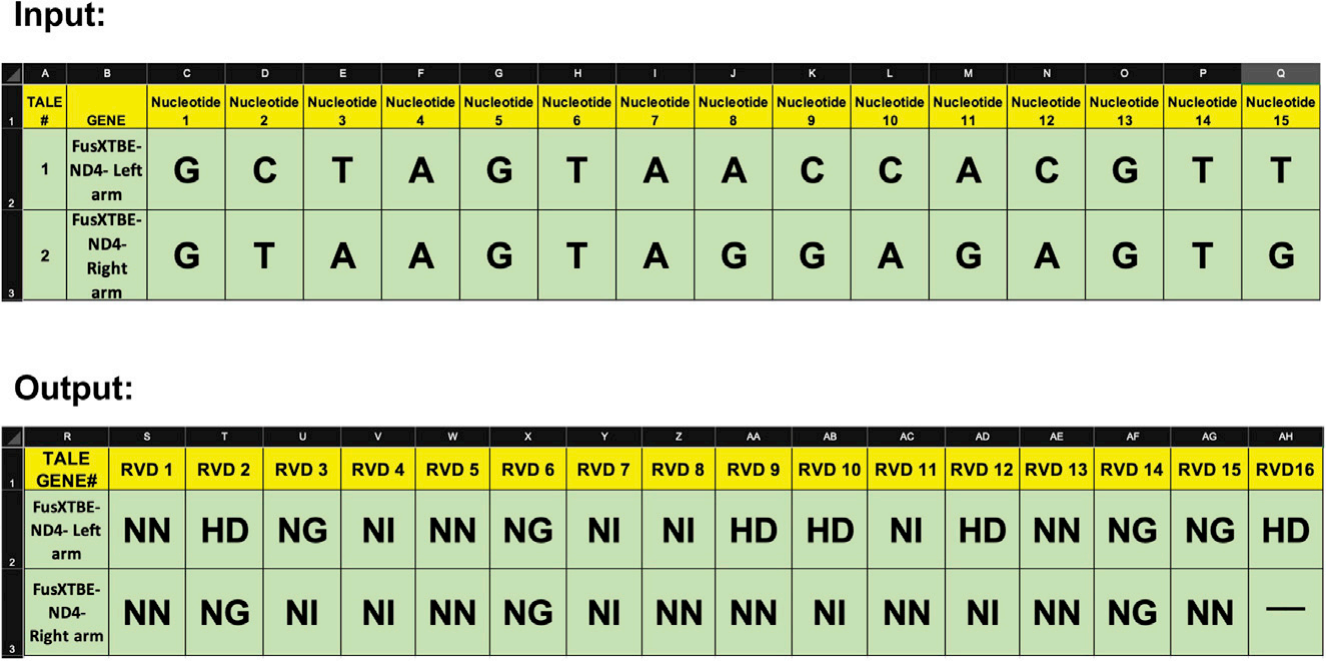
Screenshots from the “FusXTBE Builder Template” The left and right binding region of *MT-ND4* locus specific FusXTBE arms were used as **“**input” (TALE#1 and TALE#2) to obtain RVDs sequence as an “output”.

**Figure 5:**
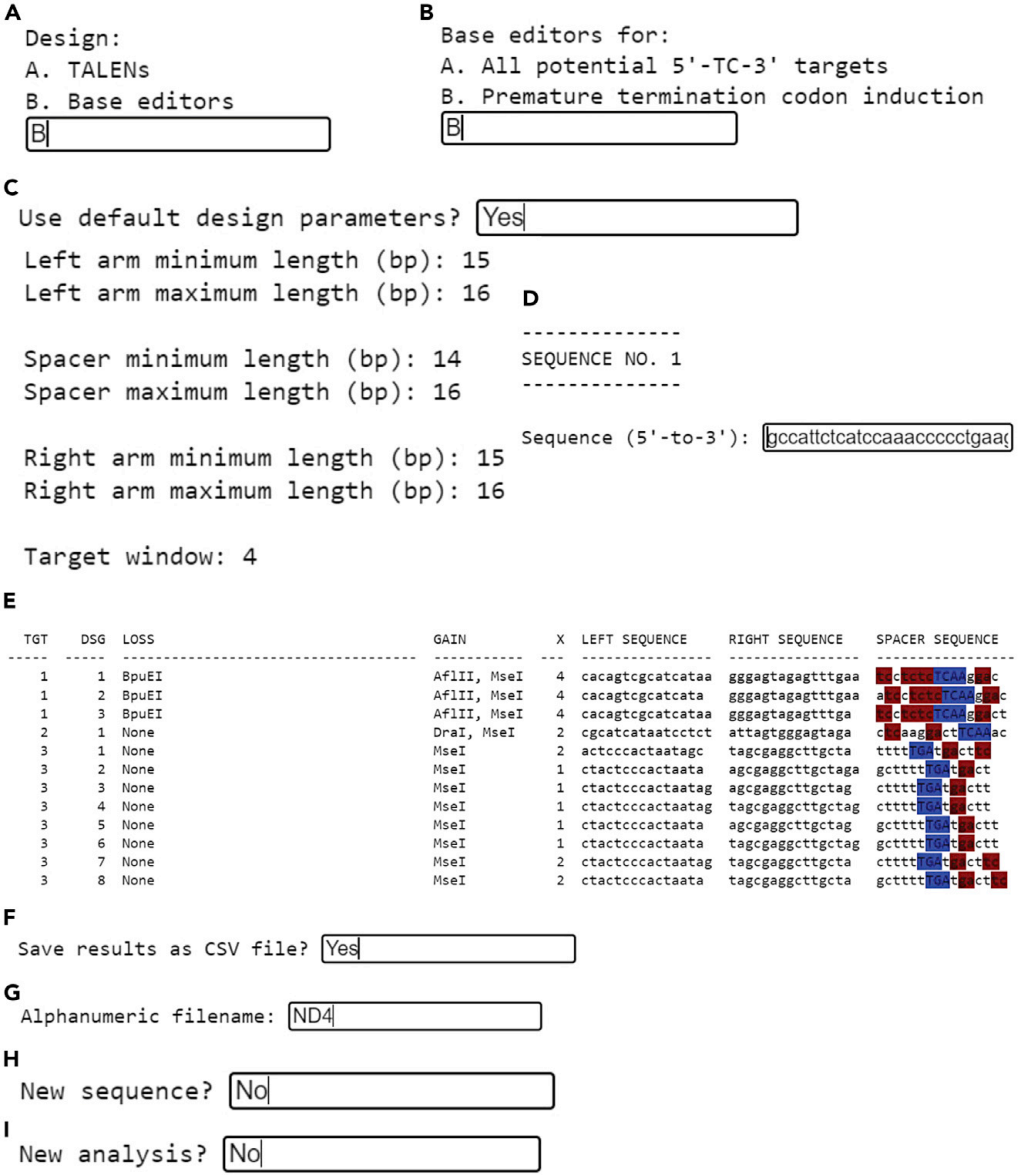
Screenshots depicting the execution of TALE Writer at setup and designing steps
  g. Choose the type of target you wish to edit (5′-TC-3′ motifs or PTC motifs only). To design FusXTBEs for premature termination codon induction, select this option by typing in ‘B’ and pressing ENTER (Figure 5B).
  h. Choose whether to use the default design parameters or your own design parameters. For FusX-based assembly, we recommend using the default parameters. Type in ‘B’ and press ENTER. The default parameters will be printed (Figure 5C).
    ***Note:*** A detailed explanation of all the inputs and outputs of TALE Writer, including the design parameters, is available at https://github.com/srcastillo/TALE-Writer/blob/main/README.md.
  i. Paste the DNA sequence you wish to screen for targets for PTC induction and find their corresponding FusXTBEs in the textbox and press ENTER.
    ***Note:*** For PTC induction, the input DNA sequence must correspond to the sense strand of a protein-coding gene and must be in-frame with the ORF of that gene. In this example, we are analyzing part of *MT-ND4* gene. This sequence is in frame with the *MT-ND4* ORF and corresponds to the PCR amplicon generated with the ND4 primers utilized in our previous report (Sabharwal et al., 2021). For FusXTBE design, TALE Writer can identify loss and gain of restriction sites after TC-to-TT editing, which can facilitate genotyping. TALE Writer identifies such sites by analyzing the entire input sequence, which is why we recommend using the DNA sequence of a PCR amplicon as the input sequence for TALE Writer (Figure 5D).
  j. A table containing all identified targets for FusXTBE-based C-to-T editing will be automatically generated (see a section of this table below). From left to right, the table contains:
    i. TGT, the target index.
    ii. DSG, the design index.
    iii. LOSS, the enzymes whose restrictions are lost after C-to-T editing.
    iv. GAIN, the enzymes whose restriction sites are gained after C-to-T editing.
    v. X, the number of potential off-target edits within the protospacer region.
    vi. LEFT SEQUENCE, the 5′-to-3′ top strand TALE binding sequence of the left arm of the FusXTBE.
    vii. RIGHT SEQUENCE, the 5′-to-3′ bottom strand TALE binding sequence of the right arm of the FusXTBE.
    viii. SPACER SEQUENCE, the 5′-to-3′ top strand sequence of the protospacer region, with the on-target PTC motif highlighted in blue, and other 5′-TC-3′ off-target sites highlighted in red (Figure 5E).
  k. To save the results, type in ‘Yes’ and press ENTER (Figure 5F).
  l. Type in an alphanumeric filename (e.g., ‘ND4’) and press ENTER (Figure 5G).
  m. If you wish to analyze a new sequence using the same parameters, type in ‘Yes’, otherwise type in ‘No’ and press ENTER (Figure 5H).
  n. If you wish to begin a completely new analysis, type in ‘Yes’, otherwise type in ‘No’ and press ENTER (Figure 5I).
  o. The saved file will be available in the Files section on the left of the Colab interface. This file can be viewed and downloaded (Figure 6A).

2. Determining the binding regions of the FusXTBE arms and converting the nucleotides to the corresponding RVD sequences.
  a. After selecting the target “cytosine” residue, the DNA-binding regions of the FusXTBE arms flanking the protospacer region (Figure 2) are designed.
    ***Note:*** Keep in mind the requirement for a 5′ terminal “thymine” nucleotide upstream of the binding region.
    ***Note:*** Try to position the target “cytosine” within the middle third of the protospacer for optimal editing (try to avoid extreme ends of the protospacer).
  b. Write the FusXTBE bound DNA sequence (position by position) of each arm in the “FusXTBE Builder Template” to generate the RVDs sequences.
    ***Note:*** For the left arm of the FusXTBE, write the top strand (5′-3′) and for the right arm write the bottom strand (5′-3′) in the “FusXTBE Builder Template” (Data S2, FusXTBE Builder Template, related to step 2). This “FusXTBE Builder Template” can generate the RVD sequences for multiple target DNA sequences.
    ***Note:*** Start with the “FusXTBE Builder Template” to add nucleotide sequences in respective cells. This Excel sheet is designed to generate the RVD sequence for targeting 15 nucleotides. Depending upon the availability of an upstream 5′-T nucleotide, FusXTBEs designed to bind 16 nucleotides instead of 15 can be made, in which the 16^th^ RVD sequence is added manually in the “FusXTBE Builder Template” (AH column). We use the following TALE cipher code: NI for A, HD for C, NN for G, and NG for T.
    Example: We designed the FusXTBE_ND4_Left Arm to bind 5′-GCTAGTAACCACGTTC-3′ (sense strand, 16 bp) and FusXTBE_ND4_Right Arm to bind 5′-GTAAGTAGGAGAGTG-3′ (antisense strand, 15 bp). After writing each nucleotide into their designated position in the “FusXTBE Builder Template”, here is the snapshot of the outcome (Figure 3). We have manually input “HD” for the last “C” nucleotide of FusXTBE_ND4_Left Arm in the AH column as it had a 16-nucleotide binding target.

3. Conversion of RVD sequences to the FusX recipe.
  Next, paste the RVD sequences generated in the previous step into the FusX recipe tool (http://www.talendesign.org/pFUXrecipeInput.php) to get the information about which plasmids from the FusX kit need to be used in the next “assembly step” to generate the required FusXTBE arms.
  Example: Below are the snapshots of the outcome for the TAL1- FusXTBE_ND4_Left Arm, and TAL2- FusXTBE_ND4_Right Arm (Figure 4).
  ***Alternatives:*** Steps 1–3 can be automated using TALE Writer. TALE Writer is a Python-based script for the computer-aided design of TALE-based technologies. Additionally, it can be used to identify potential target sites for TC-to-TT base editing in mitochondrial genomes (Sabharwal et al., 2021). Here, we provide a step-by-step explanation on how to use TALE Writer, targeting the *MT-ND4* gene as an example. To make TALE Writer available to the broader scientific community, we use Google Collaboratory (Colab), a product from Google Research. Colab requires no setup and provides free access to computing resources.
  a. To access TALE Writer, go to https://colab.research.google.com/github/srcastillo/TALE-Writer/blob/main/TALE_Writer_Colab.ipynb (you must be logged in with a Google Account to use this resource). You will be taken to the TALE Writer Colab interface.
  b. Scroll down to the “Setup” section and follow the on-screen instructions.
  c. Install third-party software by pressing the Play button to the left of the corresponding instruction.
    ***Note:*** Third-party software installation takes place in the cloud and not on your computer. If you get the message “Warning: This notebook was not authored by Google”, click on “Run anyway”.
  d. Define libraries and functions by pressing the play button to the left of the corresponding instruction.
  e. Once third-party software installation is done, and libraries and functions are defined, scroll down to the “Design” section, and click on the play button to the left of the corresponding instruction.
  f. Choose the type of TALE-based technology you wish to design (TALENs or base editors). For this protocol, to design FusXTBEs, select the base editors option by typing in ‘B’ and pressing ENTER (Figure 5A).

**Figure 6:**
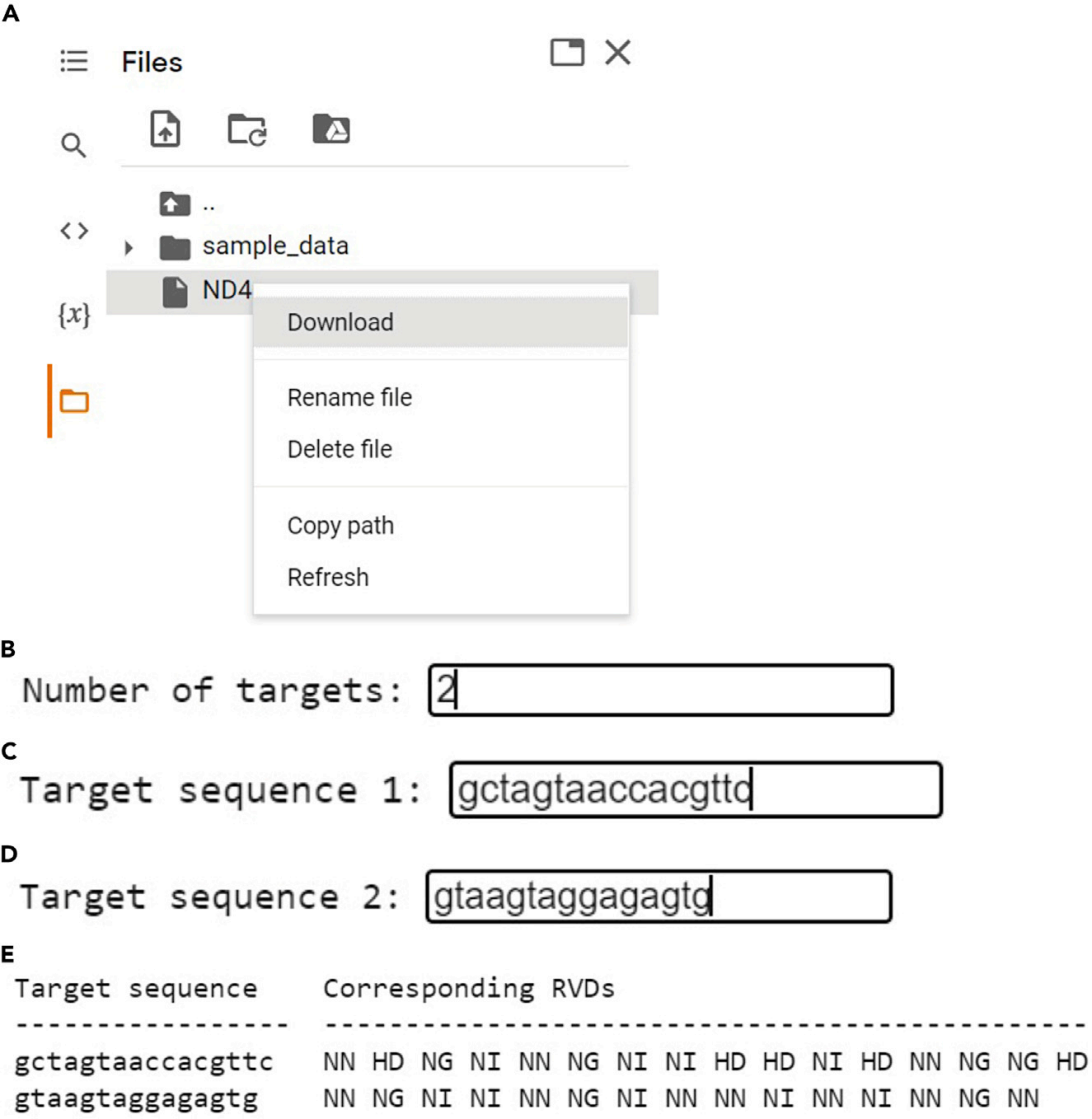
Screenshots depicting how to save the of TALE Writer output and execution at assembly step
  p. From the output table depicted in step ‘j’, a specific design or set of designs for their subsequent FusX-based assembly must be chosen. Choosing a specific design will depend on the experimental needs of the user.
  q. To obtain the FusX recipes of the chosen FusXTBEs, scroll down to the Assembly section and follow the on-screen instructions. Click on the Play button.
  r. Specify the number of targets you wish to find the corresponding RVDs for. For example, we have two targets (left arm and right arm of the ND4 FusXTBE). Type in ‘2’ and press ENTER (Figure 6B).
  s. Paste the target sequence of the left arm and press ENTER. In this example, the TALE binding sequence of the left arm of the ND4 FusXTBE is 5′-GCTAGTAACCACGTTC-3′ (Figure 6C).
  t. Paste the target sequence of the right arm and press ENTER. In this example, the TALE binding sequence of the right arm of the ND4 FusXTBE is 5′-GTAAGTAGGAGAGTG-3′ (Figure 6D).
  u. A table with the target sequences and their corresponding RVDs will be automatically generated (Figure 6E).
  v. Paste the RVD sequences generated in the previous step directly into (http://www.talendesign.org/pFUXrecipeInput.php) to get the FusX recipe.

**Figure 4:**
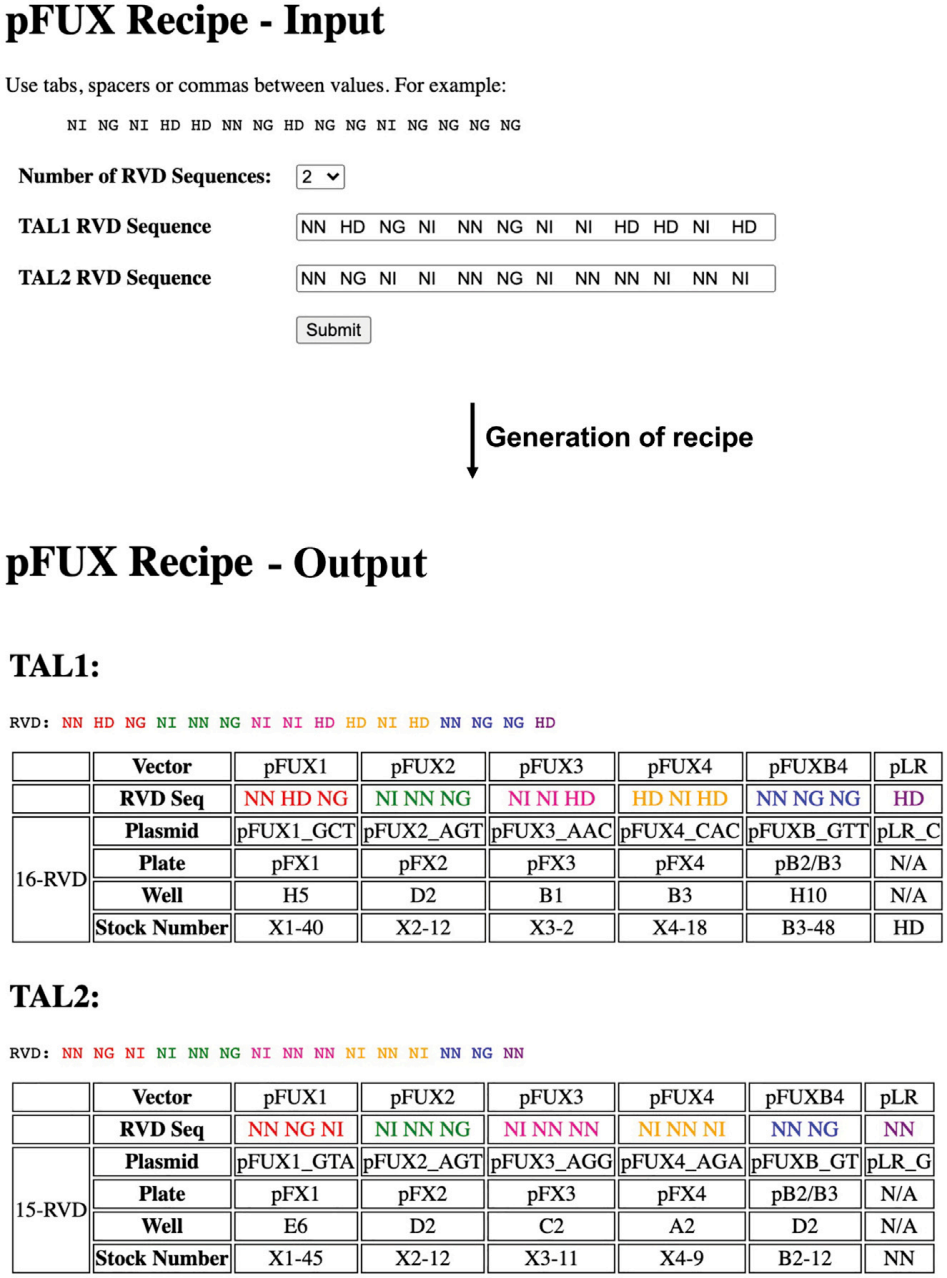
Example of conversion of RVD sequences to FusX recipe Screenshots of the input fields in the tale design site to obtain the respective plasmid’s FusX recipe for the assembling step from the RVD sequences. Here we are using the *MT-ND4* locus as an example. Note that ‘pFUX’ and ‘pFusX’ terminology are used interchangeably.
4. Golden gate assembly protocol.
  a. Set-up the assembly reaction by adding the following reagents in a PCR tube.

**Table:** 

Reagents	Amount	Final concentration
Receiving plasmid (75 ng/μL)	1 μL	75 ng
pFusX1 (50 ng/μL)	1 μL	50 ng
pFusX2 (50 ng/μL)	1 μL	50 ng
pFusX3 (50 ng/μL)	1 μL	50 ng
pFusX4 (50 ng/μL)	1 μL	50 ng
pFusB2/B3 (50 ng/μL)	1 μL	50 ng
pLR HD/NG/NI/NN (50 ng/μL)	1 μL	50 ng
BsmBI (20,000 U/mL)	0.75 μL	15 units
NEBuffer r3.1 (10×)	1 μL	1×
Nanopure water	1.25 μL	N/A
Total Volume	10 μL

    ***Note:*** Any of the following plasmids (FusXTBE-G1397-DddA_tox__Nterm, FusXTBE-G1397-DddA_tox__Cterm, FusXTBE-G1333-DddA_tox__Nterm, FusXTBE-G1333-DddA_tox__Cterm) can be used as the receiving plasmid.
    ***Note:*** The plasmid stock concentration of receiving plasmid and FusX plasmids should be adjusted to 75 and 50 ng/μL respectively for easy pipetting.
    ***Note:*** Add the FusX kit components according to their “Stock number” to the reaction.
    ***Note:*** The schematic representation of the workflow of the single Golden Gate cloning step was represented in Figure 1A.
    Example: We have put RVDs specific to the left and right arms of the *MT-ND4* target into the two receiving plasmids: 1. FusXTBE-G1397-DddA_tox__Nterm, and 2. FusXTBE-G1397-DddA_tox__Cterm. Thus, a total of four different plasmids were generated, which allowed to try out two different orientations (see expected outcomes).

**Table:** 

Arms being synthesized Component	FusXTBE-ND4- left arm-G1397-DddA_tox__Cterm	FusXTBE-ND4-right arm-G1397-DddA_tox__Nterm	FusXTBE-ND4- left arm-G1397-DddA_tox__Nterm	FusXTBE-ND4-right arm-G1397-DddA_tox__Cterm
Receiving plasmid (75 ng/μL)	FusXTBE-G1397-DddA_tox__Cterm (1 μL)	FusXTBE-G1397-DddA_tox__Nterm (1 μL)	FusXTBE-G1397-DddA_tox__Nterm (1 μL)	FusXTBE-G1397-DddA_tox__Cterm (1 μL)
pFusX1 (50 ng/μL)	#40 (1 μL)	#45 (1 μL)	#40 (1 μL)	#45 (1 μL)
pFusX2 (50 ng/μL)	#12 (1 μL)	#12 (1 μL)	#12 (1 μL)	#12 (1 μL)
pFusX3 (50 ng/μL)	#2 (1 μL)	#11 (1 μL)	#2 (1 μL)	#11 (1 μL)
pFusX4 (50 ng/μL)	#18 (1 μL)	#9 (1 μL)	#18 (1 μL)	#9 (1 μL)
pFusB2/B3 (50 ng/μL)	B3#48 (1 μL)	B2#12 (1 μL)	B3#48 (1 μL)	B2#12 (1 μL)
pLR HD/NG/NI/NN (50 ng/μL)	HD (1 μL)	NN (1 μL)	HD (1 μL)	NN (1 μL)
BsmBI (20,000 U/mL)	0.75 μL	0.75 μL	0.75 μL	0.75 μL
NEBuffer r3.1 (10×)	1 μL	1 μL	1 μL	1 μL
Nanopure water	1.25 μL	1.25 μL	1.25 μL	1.25 μL
Total Volume	10 μL	10 μL	10 μL	10 μL
  b. Incubate the reaction mixture at 55°C for 1 h.
  c. Add the following reagents to all tubes.

**Table:** 

Reagent	Amount	Final concentration
Esp3I (10 U/ μL)	0.5 μL	5 units
T4 DNA ligase buffer (10×)	1.5 μL	1×
T4 DNA ligase (400,000 U/mL)	0.5 μL	200 units
ATP (25 mM)	0.5 μL	0.83 mM
Nanopure water	2 μL	N/A
Total Volume	15 μL

    **CRITICAL:** Always use fresh ATP or fresh ligase buffer as ATP breaks down during freeze-thaws. Make aliquots of freshly received ATP and store them at −20°C for single use.
  d. Run the following cycle in PCR machine.

**Table:** 

Temperature	Time	Number of cycles
16°C	1 h	1
37°C	7 min	6
16°C	15 min	
37°C	10 min	1
50°C	5 min	1
80°C	5 min	1
12°C	Hold

    ***Note:*** Esp3I is an isoschizomer of BsmBI. It is active at 37°C (instead of 55°C).
  e. Add 0.5 μL of Plasmid-Safe ATP-Dependent DNase and 0.5 μL of 25 mM ATP to each tube.
  f. Incubate at 37°C for 1 h.
  g. Inactivate the DNase by incubating at 70°C for 30 min.
    **Pause point:** The assembling protocol can be paused here, and the samples can be stored at −20°C for next day.
  h. Transform 2.5 μL of assembly product into 25 μL of DH5α competent cells (any subcloning efficiency DH5α competent cell should work fine).
  i. Plate 50–100 μL of transformed culture onto LB-kanamycin agar plates previously supplemented with 40 μL of X-Gal and 40 μL of IPTG for blue/white screening (see materials and equipment). Incubate at 37°C for 12–18 h.
    ***Note:*** After the heat shock during bacterial transformation, either SOC or LB liquid media can be used to recover the cells. Recovery time should be 1 h.
    ***Note:*** The FusXTBE receiving plasmids contain a kanamycin resistance gene.
    **Pause point:** Optionally, the plates having colonies can be stored at 4°C for a few days before colony PCR.

5. Validation of FusXTBE plasmid assembly.
  a. Run a colony PCR to verify the final FusXTBE plasmids. For this, pick 4–8 white colonies from each plate.
    ***Note:*** Only individual white colonies have to be picked for the colony PCR. Sample the bacterial colony either with a sterile toothpick or a pipette tip, briefly dip in the colony PCR master mix (do not mix around vigorously) and subsequently inoculate the same toothpick or tip in a culture tube containing LB media supplemented with kanamycin. Alternatively, some portion of the colony can be used in colony PCR and the remaining of the colony can be used for inoculation in LB media after colony PCR screening.
  b. Set up the following PCR with MyTaq DNA Polymerase.

**Table:** 

Reagent	Amount
5× MyTaq buffer	3 μL
Forward primer- Tal-F1 (10 μM)	0.75 μL
Reverse primer- Tal-R1 (10 μM)	0.75 μL
MyTaq polymerase (5 U/μL)	0.1 μL
Nanopure water	14.2 μL
Colony	N/A
Total Volume	15 μL
  c. Run the following PCR cycle.

**Table:** 

Steps	Temperature	Time	Cycles
Initial Denaturation	95°C	2 min	1
Denaturation	95°C	30 s	35 cycles
Annealing	55°C	20 s	
Extension	72°C	2.5 min	
Final extension	72°C	5 min	1
Hold	12°C	forever
  d. Run all the PCR samples on a 1% gel and look for a smear pattern (Figure 7A shows a representative agarose gel for this step).
    ***Note:*** In our study, we used Meridian Bioscience DNA polymerase. Other Taq-based polymerases can also be used, following their corresponding protocols.
    ***Note:*** If there is a high proportion of white to blue colonies, then probability of true positive colonies is high. We recommend screening at least 4 white colonies per assembly.
    ***Note:*** The smeary ladder results from amplicon slips from one repeat region to another during denaturation and renaturation, especially when the extension is incomplete. These partial products can bind at a different RVD location due to the repetitive nature of TALE repeats. The partial products bind to either full-length or partial products in different locations to make longer or shorter end products. Once they are made, they can be readily amplified by the standard primers, which contributes to the laddering. Negative colonies result in a single band due to the absence of TALE repeats (Figure 7A, lanes 5 and 6).

**Figure 7:**
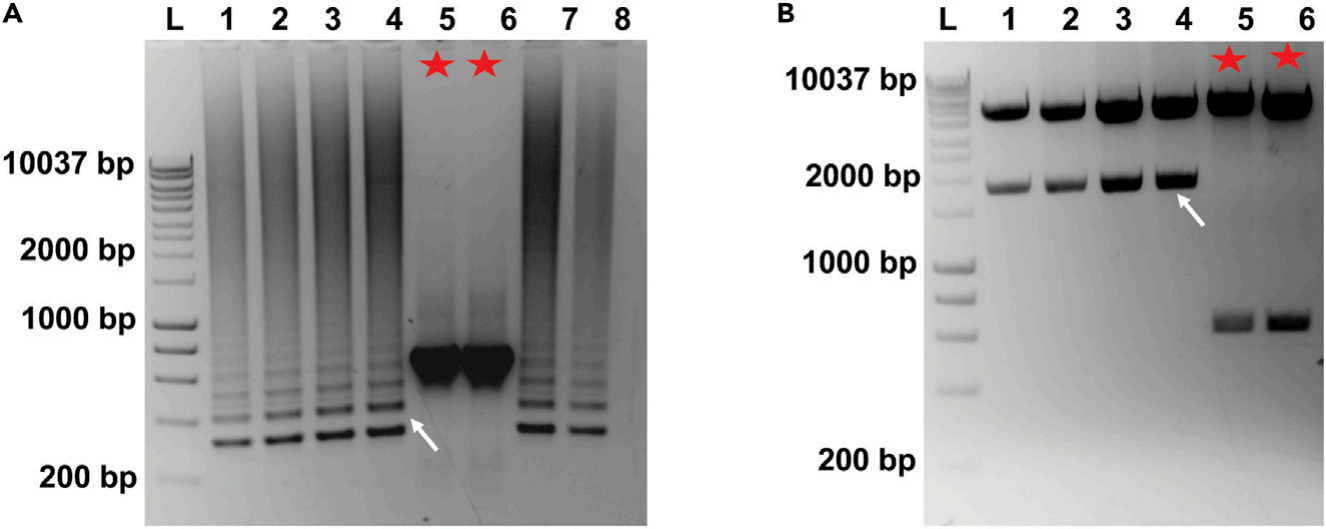
Validation of correct FusXTBE plasmid assembly (A) Representative gel image from colony PCR showing the smear pattern from the correctly assembled clones. Lanes marked by star represents the clones with unsuccessful assembly reaction and should be excluded for further experiments. (B) Gel image showing the expected restriction digestion pattern (an insert of ∼1,750 bp) for the correct clones. The star marked lane represents how the restriction digestion of the wrong clones would look.
  e. Culture one or two confirmed colonies for 14–16 h, in LB media supplemented with kanamycin.
  f. Purify the plasmid DNA by using a plasmid isolation kit/protocol.
    ***Note:*** We isolate plasmid DNA with the QIAprep Spin Miniprep Kit (QIAGEN). The supplier’s protocol should be followed during plasmid DNA purification. The extraction of the plasmid can be scaled up using a QIAGEN Plasmid Maxi Kit (Cat#12163).
    ***Alternatives:*** Other plasmid extraction kits, such Monarch Plasmid Miniprep Kit (NEB Cat. No. T1010S) can also be used.
  g. Set up the following diagnostic digest reaction for verification of the isolated plasmids.

**Table:** 

Component	Volume	Final concentration
Plasmid	X μL	500–600 ng
SphI (20,000 U/mL)	0.5 μL	10 units
XbaI (20,000 U/mL)	0.5 μL	10 units
NEB rCutSmart buffer (10×)	2.0 μL	1×
Nanopure water	X μL	N/A
Total Volume	20 μL
  h. Incubate the digestion mixture at 37°C for 1–2 h.
  i. Run all the digested samples on a 1% agarose gel. Look for a band corresponding to ∼1,750 bp (Figure 7B depicts a representative diagnostic digest).
  j. Measure the plasmid DNA concentration using a NanoDrop UV-Vis spectrophotometer or a similar method.
  k. Prepare the plasmid samples for Sanger sequencing, using the TAL-F1 and TAL-R1 primers. Many sequencing services are available, we use Genewiz (https://www.genewiz.com/).
  l. Perform the sequence confirmation analysis of the FusXTBE plasmids obtained by chromatogram/fasta sequence from Sanger sequencing either by translating the amplicon (unorthodox but easy to perform) or by making the *in silico* plasmids for sequence alignment.
    i. *Translating the sequence reads:* After getting the sequencing results back, use ExPASy Translate (http://web.expasy.org/translate/) to convert the nucleotide sequences into their corresponding amino acid sequences. Subsequently, search for the “VVAIAS“ consensus amino acid sequence, which appears immediately before each RVD.
      ***Note:*** The ExPASy Translate tool outputs amino acid sequences for all six possible reading frames. Using the sequence file generated by sequencing with the forward primer (TAL-F1), the sequence of interest will be within the first three frames; using the reverse primer (TAL-R1), the sequence of interest will be within the last three frames.
      ***Note:*** You should observe 32 amino acids in between the RVDs. A good sequencing result gives between 8-9 RVDs. Both the forward and reverse primer-generated sequencing files should be used to validate all the RVDs of a plasmid.
    ii. *In silico design of FusXTBE plasmids*: Use the SnapGene software to build the *in silico* FusXTBE plasmids. Open the correct receiving plasmid and go to Actions > Restriction and Insertion Cloning > Insert Multiple Fragments. To build the plasmid, use the appropriate FusX library plasmids according to the FusX recipe. In the SnapGene interface, cut every plasmid with BsmBI. Subsequently, Sanger sequencing files can be aligned with the resulting FusXTBE plasmid maps for cloning confirmation.
      ***Note:*** The FusX library plasmids can be downloaded one by one from Addgene (https://www.addgene.org/kits/ekker-fusx/). The pLR plasmids can be downloaded from Addgene as well (https://www.addgene.org/Daniel_Voytas/).
  m. The sequence-verified plasmid samples should be used for future experiments.
    **Example:** Here, we are showing the translation of Sanger sequencing reads of the FusXTBEs for *MT-ND4* (left and right arms, Figure 8).
    **Pause point:** The base editing plasmids can be stored at −20°C for long term storage and can be used for future transfection experiments.

**Figure 8:**
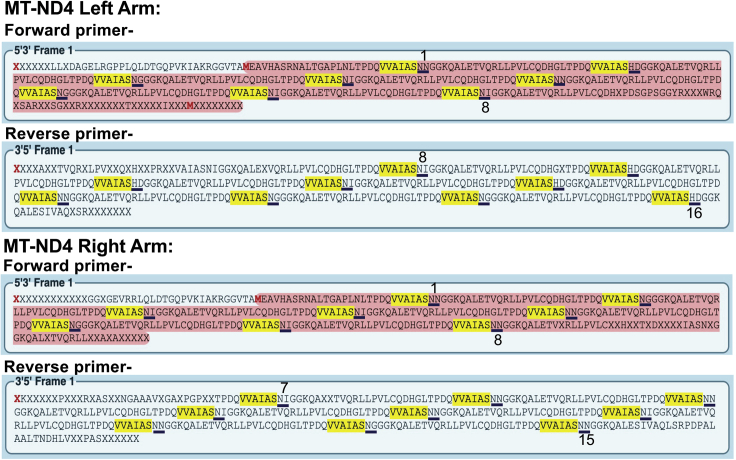
Representative figure showing the “Expasy translate” output This figure shows the translation of fasta sequence obtained from Sanger sequencing of *MT-ND4* specific FusXTBE left and right arm as an example. Sequencing by both the forward and reverse primers is necessary to check all the RVDs. The underlined amino acids represent the RVDs.

### Cell culture and transfection of FusXTBE arms

**Timing: ∼1 week**

6. Cell culture.
  The protocol described below provides the steps for maintaining HEK293T cells, including how to start a cell culture from frozen vials of cells in cryogenic storage. Similar procedures can be used for HT1080 cells as well.
  a. Thaw cryopreserved cells by putting the cryogenic vials in a 37°C water bath. Spray the thawed vial with 70% ethanol and wipe carefully to reduce the possibility of contamination. Transfer thawed liquid contents into a 10 mL conical tube prefilled with 9 mL fresh complete culture medium (DMEM supplemented with 10% fetal bovine serum and 1% Pen-Strep).
  b. Collect cells for plating.
    i. Centrifuge at 500 × *g* for 5 min, discard the supernatant, and resuspend the cells in 1 mL of fresh DMEM supplemented with 10% fetal bovine serum and 1% Pen-Strep.
    ii. Plate ∼1–2 × 10^6^ cells in a T75 flask and incubate cultures at 37°C.
  c. Passage the HEK293T cells upon 80%–90% confluency (it usually takes 4–6 days). Subculture ratio is 1:10.
    ***Note:*** Culture medium should be prewarmed to 37°C before use.

7. Cell preparation for transfection.
  a. Cell dissociation before plating.
    i. Discard culture medium and subsequently rinse the adhered cells with PBS. Aspirate PBS and add 3 mL 0.25% Trypsin solution (with EDTA) to the T-75 flask and incubate at 37°C for ∼5 min.
    ii. Tap the flask gently on the side to dislodge the cells completely and add 7 mL of DMEM to the flask to deactivate the Trypsin. Transfer the cells to a 15 mL conical tube (Tube A).
    iii. Count the cells/mL by using a hemacytometer.
      ***Alternatives:*** Automated cell counters such as ThermoFisher Scientific Countess 3 FL Automated Cell Counter (Catalog# AMQAF2000) can be used for cell counting.
  b. Seeding of cells at a suitable density.
    i. Typically, for the base editing experiments in a 6-well plate, take 18 mL of DMEM in a 50 mL conical tube and add media from tube A containing ∼1.8 × 10^6^ cells. Mix the sample gently and transfer about 3 mL of media into each well (∼0.3 × 10^6^ cells/well). Incubate cells at 37°C for 14–16 h in 5% CO2 incubator. The number of 6-well plates can be scaled up according to the needs of the experiments.
    ii. By the next day, the cells should be at 70%–80% confluent in each well and ready for transfection.
      ***Note:*** To save time, cell culture and seeding can be started ahead of time so that plasmids can be transfected immediately after sequence verification.
      ***Note:*** Alternatively, cells can be seeded on 24 or 48-well plates. Seeding density should be adjusted accordingly.

8. Transfection with FusXTBE plasmids.
  The following steps describe the detailed procedures for using Lipofectamine 3000 to transfect HEK293T cells with the FusXTBE left and right arms plasmid DNA.
  a. Add 1.2 μg (0.6 μg per arm) of FusXTBE plasmid DNA to a microcentrifuge tube, adjust the volume to 122 μL with Opti-MEM and add 3 μL of P3000 Reagent.
  b. Add 120 μL Opti-MEM and 5 μL lipofectamine 3000 to a separate microcentrifuge tube.
  c. Mix the contents of both tubes thoroughly by pipetting up and down. Spin briefly.
  d. Incubate the transfection mix at room temperature (∼25°C) for 10–15 min.
  e. Add the transfection mix to the cells dropwise, swirl the plate to evenly distribute the mixture, and incubate the cells for at 37°C.
  f. After 10 h of transfection, exchange media with fresh culture medium.
    ***Note:*** The aforementioned transfection protocol is suitable for a single well from a 6-well plate (with a surface area of 9.6 cm^2^). If larger or smaller wells are used, the recipe should be adjusted accordingly.
  g. Harvest cells 4–6 days post transfection for genomic DNA isolation.
    **Pause point:** The cells can be stored at −80°C until genomic DNA isolation.
    ***Note:*** Transfection of each pair of FusXTBE plasmids should be done in triplicate.
    ***Note:*** Untransfected control (wild type) should be set up and cells should be collected. We recommend setting up a positive transfection control by transfecting with a GFP plasmid for easy transfection confirmation.
    ***Note:*** The amount of plasmid DNA used may need to be decreased or increased to achieve the best editing efficiency.

### Sequencing readout of C-to-T editing

**Timing: 2 days**

This final section will describe the genomic DNA isolation from the cells, followed by genotyping to interrogate for C-to-T edits in the protospacer region.

9. Mitochondrial DNA isolation.
  a. 4–6 days post transfection aspirate the media, wash with 1 mL of PBS and trypsinize the cells by adding 300 μL trypsin to each well.
  b. Add 300 μL of DMEM and transfer the contents to 1.5 mL tubes.
  c. Spin the tubes at 300 × *g* for 5 min. Discard the supernatant and aspirate the residual media from the tubes.
  d. Subsequently, we isolate the genomic DNA from the cells by using DNeasy Blood & Tissue Kit (QIAGEN) following the manufacturer’s instructions. (DNeasy Blood & Tissue Handbook).

***Alternatives:*** Different genomic DNA isolation kits like ThermoFisher’s PureLink Genomic DNA Mini Kit (Cat#K182001) or NEB’s Monarch Genomic DNA Purification Kit (Cat#T3010S) can be used.

**Pause point:** Extracted DNA can be stored at −20°C until use.

10. Validation of C-to-T editing.
  a. To validate C-to-T editing, perform PCR to amplify the region around the protospacer. Prepare the PCR and mix by pipetting up and down 5–10 times.

**Table:** 

Reagent	Amount
Q5 High-Fidelity 2× Master Mix	12.5 μL
Forward primer- (10 μM)	1.25 μL
Reverse primer- (10 μM)	1.25 μL
Genomic DNA isolated in previous step (50–100 ng)	1 μL
Nanopure water	Up to 25 μL
Total Volume	25 μL
  b. Run the following PCR cycle.

**Table:** 

Steps	Temperature	Time	Cycles
Initial Denaturation	98°C	30 s	1
Denaturation	98°C	10 s	35 cycles
Annealing	Tm- 5°C	20 s	
Extension	72°C	30 s	
Final extension	72°C	2 min	1
Hold	12°C	forever

    ***Alternatives:*** Different high-fidelity polymerases, such as NEB’s Phusion High-Fidelity DNA Polymerase enzyme (Cat. No. M053S) or ThermoFisher’s AccuPrime *Taq* DNA Polymerase (Cat. No. 12346086) can be used.
  c. Extract PCR-amplified target DNA of each sample from the 1% agarose gel with QIAquick Gel Extraction Kit from QIAGEN (Cat#28706).
    ***Alternatives:*** Different gel extraction kits, such as NEB’s Monarch DNA Gel Extraction Kit (Cat#T1020L) can be used. Alternatively, PCR clean up kits can be used if only a single, clean amplicon is present.
    ***Optional:*** Measure DNA concentration using a NanoDrop UV-Vis spectrophotometer.
    **Pause point:** PCR amplicons can be stored at −20°C until further use.
  d. Submit the DNA sample for Sanger sequencing, using the forward or reverse PCR primer as the sequencing primer.
  e. As a negative control, sequence DNA from wild type cells (with no FusXTBE transfection).
    **Pause point:** Analysis of Sanger sequencing data can be conducted at any time.
  f. Evaluate C-to-T base editing efficiencies from Sanger sequencing results using EditR (Kluesner et al., 2018).
    i. Obtain Sanger sequencing reads in .ab1 extension format.
      ***Optional:*** Directly visualize the Sanger sequencing traces using a program such as SnapGene.
    ii. Visit the EditR website (https://moriaritylab.shinyapps.io/editr_v10/).
    iii. Enter the protospacer region as the gRNA sequence for analysis.
    iv. Upload the .ab1 sequencing file for the intended sample under “Upload .ab1 File”.
    v. If the .ab1 sequencing file is generated by using the reverse primer in Sanger sequencing sample, tick the “Guide sequence in reverse complement” option.
    vi. Press the “Predicted Editing” button in the upper panel section to evaluate C-to-T base editing at the target site.
    vii. The editing report can be downloaded by clicking on “Download Report”.
      ***Note:*** To evaluate the off-target edits across the amplicon, 10–15 nucleotide length regions around the protospacer region can be entered as gRNA sequence for analysis.
      ***Note:*** Usually, we observe baseline heteroplasmy using EditR. We recommend confirming the edits by doing next generation sequencing to assess the edited frequency of TC-to-TT in each allele after observing significant base editing from Sanger sequencing data. We use the NGS: AMPLICON-EZ service by Genewiz.
      ***Note:*** At this time, we don’t recommend the regular passaging of edited cells as the percentage of heteroplasmy may go down. For any down-streaming experiment that requires edited cells carrying a specific level of heteroplasmy, the expectation is the fresh preparation of cells after editing without long-term passaging of the cells.

## Expected outcomes

**Figure 9:**
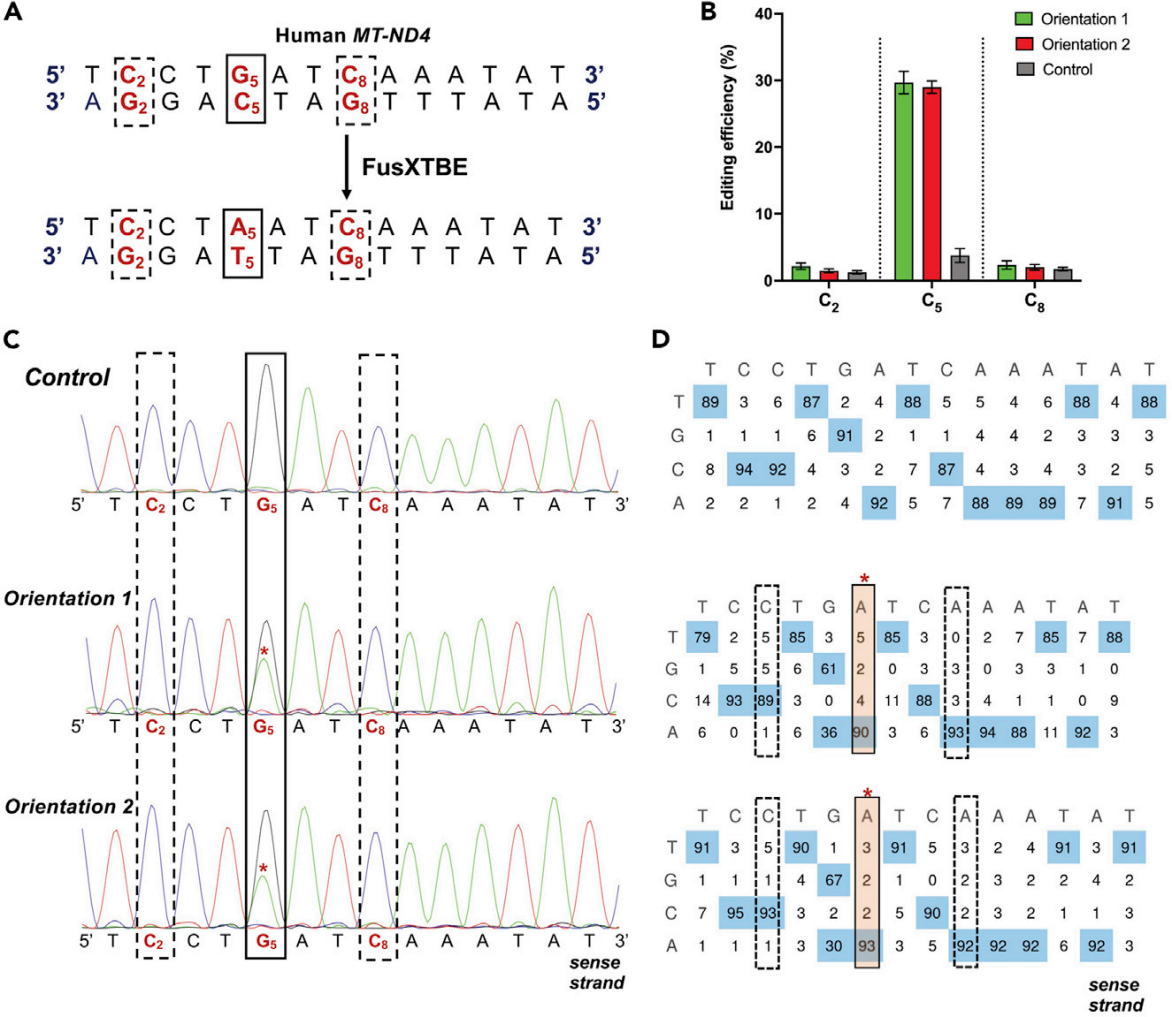
Representative figure showing the C-to-T editing activity of FusXTBE system (A) Potential target sites in the protospacer region of the targeted human *MT-ND4* locus. The solid square representing the target nucleotide for base editing and the dotted squares representing the cytosine nucleotides are amenable to base editing within the protospacer but not desired. (B) Editing efficiency of the FusXTBE when G1397 split orientations were tried independently and assessed by Sanger sequencing. Green and red colored column represent editing efficiency when FusXTBE-ND4-Left arm-G1397-DddA_tox__Cterm/FusXTBE-ND4-Right arm-G1397-DddA_tox__Nterm and FusXTBE-ND4-Left arm-G1397-DddA_tox__Nterm/FusXTBE-ND4-Right arm-G1397-DddA_tox__Cterm combinations were tried, respectively. Grey colored columns represent the wild type heteroplasmy in the target locus in the untransfected cells. Error bars are represented as standard error of the mean. No significant difference was observed in the editing efficiencies between the two orientations, and we didn’t observe any undesired base editing. (C) Representative chromatogram of the control (untransfected) and cells transfected with both the orientation of G1397 split-FusXTBE. The solid square denotes the cytosine nucleotide, which was edited, and the dotted squares denote the cytosine nucleotides which were not edited even if favored for the base editing events. (D) Editing table plots for the corresponding chromatograms. Chromatograms and editing table plot were obtained using EditR.

Using this protocol, we were able to introduce TC-to-TT edits in human mitochondrial DNA. Direct visualization of the Sanger sequencing traces (.ab1 files) or data analysis via EditR should show two dominant nucleotides (cytosine/thymine or guanine/adenine depending on the DNA strand) at the target site. We observe two peaks because: it is very difficult to achieve 100% transfection efficiency, and each cell may contain thousands of mitochondrial genomes and targeting each mtDNA for editing may not be feasible. In contrast, samples with no FusXTBE treatment (negative controls) should show a single dominant nucleotide peak of either cytosine or guanine depending on the DNA strand (Figure 9). This protocol has been tested primarily in HEK293T cells, but it also works well in HT1080 cells. This protocol can also be applied to other adherent cell lines; however, the editing efficiency would differ from cell line to cell line. Please see more detailed analysis and discussion in the original manuscript (Sabharwal et al., 2021).

Example: As previously mentioned for the *MT-ND4* editing experiment, we tried 2 different orientations of G1397 split variants of FusXTBE receiving plasmids (Table 2). The genomic DNA was isolated and the *MT-ND4* locus was amplified by using the ND4-F1 and ND4-R1 primer pair.

**Table 2:** Orientations of FusXTBE arms for the TC-to-TT editing in *MT-ND4* locus

Orientation/condition	Left arm binding FusXTBE	Right arm binding FusXTBE
1	FusXTBE-ND4- Left arm-G1397-DddA_tox__Cterm	FusXTBE-ND4-Right arm-G1397-DddA_tox__Nterm
2	FusXTBE-ND4- Left arm-G1397-DddA_tox__Nterm	FusXTBE-ND4-Right arm-G1397-DddA_tox__Cterm

We used forward primer (ND4-F1) for Sanger sequencing sample preparation and subsequently, the .ab1 files were used as inputs for EditR to estimate the editing efficiencies for each orientation. For the wild type samples (negative controls), we did not observe any significant C-to-T editing. In contrast, for orientations 1 and 2 we observed 30%  ±  4% and 29%  ±  2% of C-to-T editing, respectively, at C_5_ (m.11922 target position)**.** We did not observe any significant editing at C_2_ (m.11919) or C_8_ (m.11925), which were undesired mutations in the context of our example experiment (Figure 9). Additionally, we have successfully tested these two orientations in HT1080 cells and observed similar editing efficiencies for both.

## Limitations

Two factors that impede the availability of each mitochondrial cytosine for base editing are: 1-the strong preference of FusXTBEs for 5′-TC motifs (however, this limitation helps introducing precise mutations in the protospacer if multiple cytosine nucleotides are present), and 2-the requirement of a “T” nucleotide at the 5′ end of the FusXTBE binding sites. Base editing efficiency can vary between loci and experiments. It may be advisable using every possible orientation and trying different protospacer lengths for a target cytosine. This iterative approach may help increasing editing efficiencies.

## Troubleshooting

### Problem 1

Few or no white colonies or more blue colonies as compared to white colonies from step 5a.

### Potential solution

Prior to the Golden Gate assembly reaction, digest the receiving plasmid for 14–16 h with BsmBI. You should observe a larger band around ∼5,100 bp and a drop-out band around ∼460 bp. Extract the larger band from the gel and use that for the assembly reaction. Use the 75 ng of the gel purified DNA in the assembly reaction.

### Problem 2

Colony PCR and restriction digestion products appear to be of the correct size, but the sequencing data shows wrong RVDs from step 5l.

### Potential solution

First, this protocol uses very repetitive DNA sequences and multiple digest/ligation reactions to produce a complete FusXTBE plasmid. Due to the nature of the component plasmids, occasionally, recombinations can occur that result in deletion or addition of a TALE repeat. In this scenario, send a second miniprep from a different positive clone for sequencing. If the problem persists, double check the different FusX plasmids used in the assembly reaction and repeat the assembly process.

### Problem 3

Not able to confirm all the 15/16 RVDs from the Sanger Sequencing data from step 5l.

### Potential solution

Usually, Sanger sequencing by using the TAL-F1 and/or TAL-R1 primers should cover all the RVDs. Otherwise, try using the primer TAL-MF (5′ CTCACACCCGATCAGGTC 3′) as the forward sequencing primer. The sequencing data from this primer will confirm the middle RVDs as it specifically binds to an RVD in the eighth position.

### Problem 4

Sanger sequencing failed or spectra are not clean from step 10f.

### Potential solution

Amplify more PCR product, purify it and submit it for sequencing. Double check the input DNA concentration required by the vendors.

### Problem 5

No detected TC-to-TT base edit from Sanger sequencing of the target locus. Expected outcomes.

### Potential solution

There are several reasons why the intended TC-to-TT change may not be observed. As mentioned previously, mitochondrial base editing efficiency is governed by the target loci. First, we recommend trying both the orientations of G1397 split variants to assess for the editing efficiency in the target locus. If the editing percentage is low or negligible then try the combination of using the G1333 split variants. Second, multiple protospacer options including the target cytosine should be probed for the cytosine residues amenable to base editing. Third, NGS should be performed to investigate low percentage of edits in each allele at target loci. Other reasons may include low plasmid transfection efficiency, poor plasmid quality, the health of the cells at the time of transfection, too high or too low cell confluency at the time of transfection, etc.

### Problem 6

Off-target genome editing. Expected outcomes.

### Potential solution

As the FusXTBE cargos are destined to mitochondria, off-target mtDNA editing can be accessed by performing whole mtDNA sequencing. In previous studies, insignificant or very low off-target editing has been observed for the TALE-based mitochondrial base editors (Mok et al., 2020; Guo et al., 2021). We also did not observe any significant off-target editing around the *MT-ND4* protospacer and only a single off target-editing around the *MT-ND2* protospacer (Sabharwal et al., 2021). One potential strategy for minimizing off-target effects is to standardize the concentration of the FusXTBE arm being delivered. However, increasing specificity by reducing the amount of FusXTBE plasmids may lead to a significant reduction in on-target editing.

## Resource availability

### Lead contact

Further information and requests for resources and reagents should be directed to and will be fulfilled by the lead contact, Stephen C. Ekker, Ekker.Stephen@mayo.edu.

### Materials availability

All the 4 receiving plasmids will be made available through Addgene soon and are available upon request. The Ekker Lab’s FusX assembly kit can be ordered through Addgene (Kit No. 1000000063). The plasmid maps for all the receiving plasmids and the “FusXTBE Builder Template” were deposited to Mendeley Data (https://doi.org/10.17632/pw24c8ndk3.2).

## Data Availability

This study did not generate any unique datasets or code.

## Supplemental information

Data S1. Receiving plasmid maps, related to step 5

Data S2. FusXTBE builder template, related to step 2
